# Visual efficacy after implantation of monofocal intraocular lens on one eye and higher-order aspheric IOL on the other eye

**DOI:** 10.1186/s12886-025-04004-z

**Published:** 2025-04-14

**Authors:** Ho Sik Hwang, Ha Rim So, Hyun Seung Kim, Eun Chul Kim

**Affiliations:** 1 Yeouido St. Mary’s Hospital, Department of Ophthalmology, The Catholic University of Korea, College of Medicine, Seoul, Korea; 2 Bucheon St. Mary’s Hospital, Department of Ophthalmology, The Catholic University of Korea, College of Medicine, Seoul, Korea; 3 Seoul St. Mary’s Hospital, Department of Ophthalmology, The Catholic University of Korea, College of Medicine, Seoul, Korea

**Keywords:** Visual efficacy, Intermediate vision, Cataract, Monofocal intraocular lens, High-aberration aspheric intraocular lens

## Abstract

**Purpose:**

To compare visual and refractive outcomes and patient satisfaction results after binocular cataract surgery with implantation of both TECNIS Eyhance ICB00 and TECNIS ZCB00 intraocular lenses in the same patient.

**Methods:**

One hundred twelve eyes from 56 patients who underwent intraocular lens implantation with Eyhance ICB00 and ZCB00 in the same patient were retrospectively enrolled. Pre-operative and post- operative uncorrected and corrected near (UNVA, CNVA), intermediate (UIVA, CIVA), distant (UDVA, CDVA) visual acuity, and depth of focus were analyzed. Satisfaction surveys were evaluated after cataract surgery.

**Results:**

There was no significant difference in the preoperative values between the Eyhance ICB00 and ZCB00 groups. At two months postoperatively, UNVA of the ZCB00 group (0.02 ± 0.03 logMAR) was significantly better than that of the Eyhance ICB00 group (0.05 ± 0.05) (*P* < 0.05). At one week, one month, and two months postoperatively, the UIVA of the Eyhance ICB00 was significantly better than that of the ZCB00 group (*P* < 0.05). At two months postoperatively, the UNVA of the Eyhance ICB00 was significantly better than that of the ZCB00 group (*P* < 0.05). In the satisfaction survey, daily activities were not limited by their vision or glare in both groups.

**Conclusion:**

Inserting a high-aberration aspheric intraocular lens(IOL) in one eye rather than inserting a monofocal IOL in both eyes is better for improving distance, intermediate, and near vision. Furthermore, patients who have Eyhance ICB00 in one eye and a monofocal IOL in the other do not experience any difficulty in their daily lives.

## Introduction

A cataract is an opacity that develops in the transparent lens. It can result in treatable vision impairment or blindness. Cataract surgery is an effective treatment for visual loss caused by lens opacity. Phacoemulsification and inserting an intraocular lens (IOL) is one of the most common operations in the ophthalmology area. With the development of cataract surgery methods, there have been many studies and developments in the use of intraocular lenses. After inserting a conventional monofocal IOL that focuses on the far side, the lens's accommodative strength is diminished or lost, necessitating the use of glasses for near tasks, which is disadvantageous. If a myopic target is chosen and there is no high astigmatism, the need for near spectacles is usually none. To overcome this problem, bifocal or multifocal intraocular lenses with two or more focal lengths have been developed [1]. With the development of such multifocal intraocular lenses, both distance and near vision can be restored after cataract surgery.

However, since not only near vision but also intermediate distance vision occupies a large part of daily life, the need for an intraocular lens to restore both near vision and intermediate distance vision has emerged. In addition, there are many cases of patients with multifocal IOLs complaining of halos, glare, and decreased contrast sensitivity after surgery compared to patients with monofocal IOLs. TECNIS Eyhance ICB00 (Johnson & Johnson Vision Care, Inc., New Brunswick, NJ, USA) was developed to restore intermediate vision after cataract surgery. Many studies have compared clinical results after surgery using the TECNIS Eyhance ICB00 and the diffractive multifocal IOL or monofocal IOL [2–6].

The purpose of this study was to compare clinical results after surgery using TECNIS Eyhance ICB00 and TECNIS ZCB00 intraocular lenses in the same patient.

## Methods

The study was approved by the Institutional Review Board (IRB) of Bucheon St. Mary Hospital (No. HC22RISI0041), and all the methods described adhered to the principles of the Declaration of Helsinki. The need for written informed consent was waived by the IRB of Bucheon St. Mary Hospital owing to the retrospective nature of this study, in accordance with the Ethical Guidelines for Medical and Biological Research Involving Human Subjects repealed from the Korean Government.

### Patients

From April 2020 to February 2022, ZCB00 was administered to the contralateral eye of patients who underwent phacoemulsification and intraocular lens implantation using Eyhance ICB00 at the Department of Ophthalmology, Bucheon St. Mary's Hospital, Catholic University of Korea. Medical records of 56 patients (112 eyes) were reviewed. For inclusion criteria, patients aged 60 years or older but less than 80 years old with regular corneal astigmatism of 1.5 diopters (D) or less before surgery were included. Those who had extreme axial lengths (> 25 mm), retinal disease, optic nerve disease, or limited vision recovery due to previous ocular trauma or ophthalmic surgery were excluded from this study.

### IOLs

Eyhance ICB00 is a one-piece acrylic aspheric refractive foldable IOL. It is 13 mm in total length, including haptics, and 6.0 mm in optics. It has a design common to ZCB00. Eyhance ICB00 is a high-aberration aspheric intraocular lens designed to have a continuous focus effect. Its depth of focus increases toward the center of the lens. It is an intraocular lens that aims to improve lifestyle intermediate distance (66 cm ~ 100 cm) visual acuity while maintaining the effect of improving distance vision at a similar level compared to conventional monofocal intraocular lenses. The design without diffraction rings and refraction zones shows results similar to that of single vision intraocular lenses in night glare and halo.

The ZCB00 is a 1-piece acrylic aspheric monofocal IOL that has a single focus. As it has an aspherical optic, it can clinically improve the visual quality and contrast sensitivity compared to a spherical IOL.

A ZCB00 IOL was implanted in the dominant eye, and an Eyhance ICB00 IOL was implanted in the non-dominant eye.

### Operative procedures

All surgeries were performed by one operator (ECK). Surgery was performed after topical anesthesia using 0.5% proparacaine hydrochloride (Alcaine®; Alcon Laboratories, Inc., Fort Worth, TX, USA). The cornea was incised with a length of about 2.75 mm. The direction of the incision was performed along the steepest axis of astigmatism by topographic examination of the cornea. A 6 mm limbal relaxing incision was performed along the opposite side of the main corneal incision especially when patients had more than 1.0 D corneal astigmatism. Continuous curvilinear capsulorhexis, hydrodissection, hydrodelineation, and phacoemulsification were performed. After phacoemulsification, a viscoelastic material was injected into the anterior chamber, and an intraocular lens was inserted into the capsular bag using an intraocular lens injector. All incisions were not sutured, and stromal hydration was performed. For postoperative eye drops, moxifloxacin four times a day and fluorometholone 0.1% four times a day for one week after surgery were used. After that, gatifloxacin four times a day and prednisolone 1% were instilled four times a day for one month. Each was then reduced to two times a day and used for another month.

### Outcome measures

Preoperative examinations included uncorrected visual acuity, corrected visual acuity, manifest refraction, automatic keratometry, IOL master 500 (Carl Zeiss Meditec AG, Jena, Germany), corneal topography examination (Pentacam, Oculus Inc., Germany), and slit lamp microscopy. The Scheimpflug system (Pentacam®, Oculus, Germany) was used to determine total corneal astigmatism. The IOL power was determined using the SRK/T, Barret Universal II, Kane, and Haigis formulas for emmetropia. Pre-operative and post- operative one week, one month, and two months, uncorrected and corrected near (UNVA, CNVA at 33 cm), intermediate (UIVA, CIVA at 66 cm), distant (UDVA, CDVA at 5 m) visual acuity, and manifest refraction were performed, and the depth of focus was measured using a defocus curve. The Snellen visual acuity was used. Visual acuity was measured by changing the spherical lens from + 1 diopter to −4 diopter in 0.5 diopter increments based on the maximum correction at a distance. The defocusing curve was obtained using the result. In the defocusing curve, the section with a visual acuity of 0.3 logMAR (minimum angle of resolution) or more was measured as the depth of focus [7, 8]. The visual acuity was converted to logMAR. The difference between the preoperative target refractive power (predicted refractive power) and the postoperative spherical equivalent was analyzed. Absolute depth of focus was obtained from those vergences [in diopters (D)], which provided VA values ≤ 0.1 logMAR. Relative depth of focus was obtained considering those vergences (in D) which provided a decay of 0.1 logMAR from to the best VA of each subject at zero vergence. Contrast sensitivity, with and without a glare source, was measured using the Contrast Sensitivity Accurate Tester (CAT-2000, Neitz, Tokyo, Japan). Higher-order aberrations were measured using wavefront aberrometry (iTrace, Tracey Technologies, Texas, USA) under mydriasis of 5 mm pupil size induced by phenylephrine tropicamide eye drops and 3 mm pupil size. Postoperative satisfaction and discomfort were investigated through the Vision-Related Quality of Life (VRQOL)-Cataract TyPE Spec questionnaire survey.

### Statistical analysis

All statistical analyses were performed using the statistical program SPSS Version 22.0 (IBM Corp., Armonk, NY, USA). Paired t-test and Wilcoxon signed rank test were used for size comparison before and after surgery. Student’s t-test and Mann–Whitney test were used for size comparison between two groups. The Kruskal–Wallis test was used for the comparison of two groups. Statistical significance was considered when the *p*-value was less than 0.05.

## Results

### Study population

Among 56 patients (112 eyes), 33 were males and 23 were females. Their average age was 67.88 years old. There was no significant difference in uncorrected visual acuity, best corrected visual acuity, manifest refraction spherical equivalent, corneal astigmatism, or axial length between the two groups (Eyhance ICB00 group and ZCB00 group) before surgery (Table 1).

**Table 1:** Preoperative characteristics of patients

**Parameter**	**Eyhance**®** ICB00**	**Tecnis**®** ZCB00**	**P value**
Eyes (n)	56		N/A
Age (y)	67.88 ± 7.51 (60 to 79)		N/A
Sex (M:F)	33: 23		N/A
UDVA (LogMAR)	0.50 ± 0.35	0.36 ± 0.42	0.057
CDVA (LogMAR)	0.37 ± 0.24	0.30 ± 0.38	0.062
UIVA (LogMAR)	0.56 ± 0.28	0.52 ± 0.32	0.375
CIVA (LogMAR)	0.48 ± 0.31	0.45 ± 0.37	0.256
UNVA (LogMAR)	0.59 ± 0.48	0.56 ± 0.39	0.421
CNVA (LogMAR)	0.46 ± 0.36	0.48 ± 0.40	0.328
MRSE (D)	−0.23 ± 2.68	−0.85 ± 2.74	0.533
CA (D)	−0.85 ± 0.46	−0.78 ± 0.62	0.245
IOL power (D)	20.18 ± 3.45	20.00 ± 3.19	0.631
Axial length (mm)	23.80 ± 1.01	23.78 ± 1.10	0.873

*CA* corneal astigmatism, *UDVA* uncorrected distance visual acuity, *CDVA* corrected distance visual acuity, *UIVA* uncorrected intermediate visual acuity, *CIVA* corrected intermediate visual acuity, *UNVA* uncorrected near visual acuity, *CNVA* corrected near visual acuity, *D* diopter, *IOL* intraocular lens, *LogMAR* logarithm of the minimum angle of resolution, *MRSE* manifest refraction spherical equivalent, *N/A* not applicable

Results are reported as mean ± SD

There were no significant differences in preoperative factors

^*^, *p* < 0.05 between groups

**Fig. 1:**
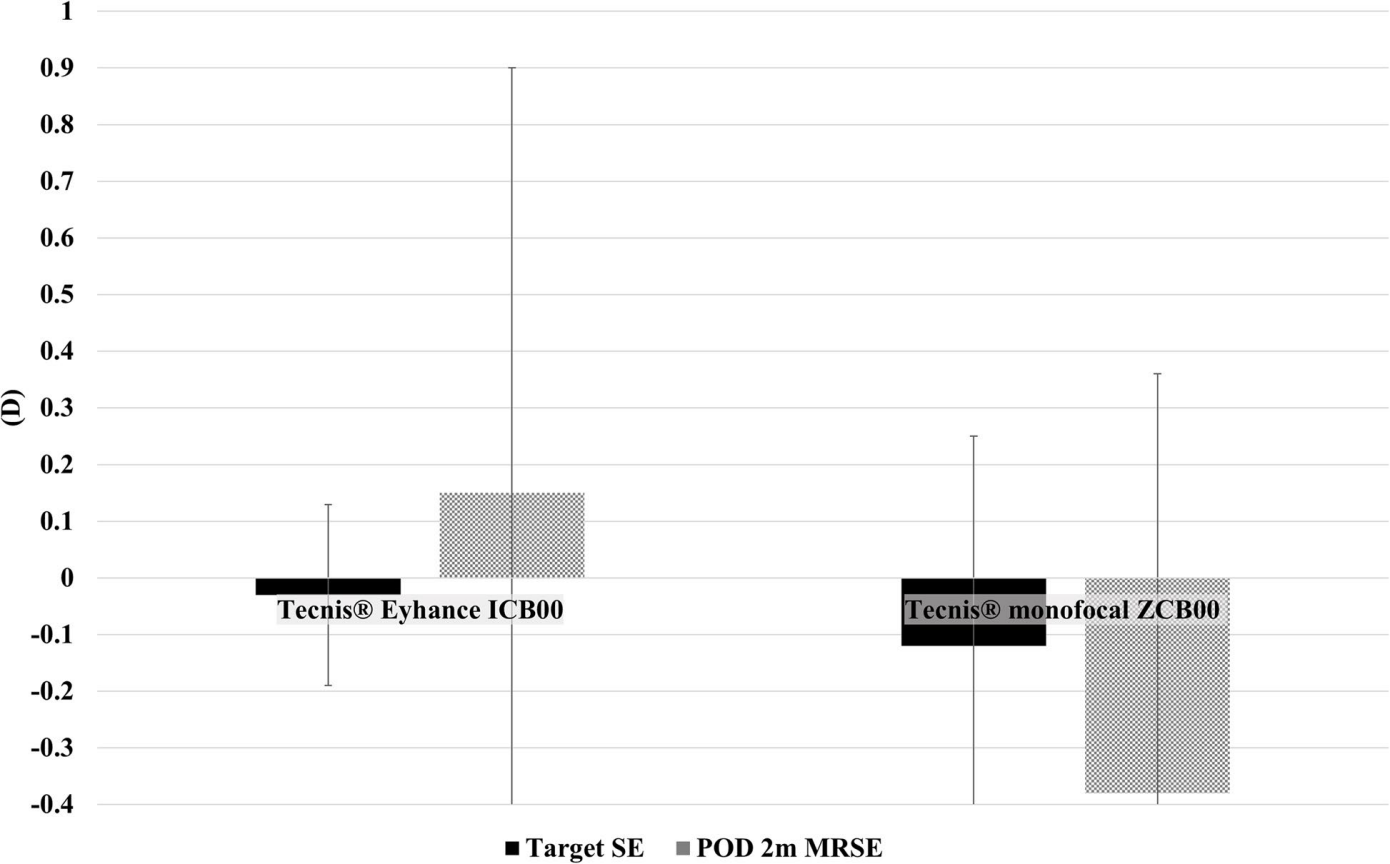
Target spherical equivalent before cataract surgery and manifest refraction spherical equivalent at two months after surgery. MRSE, manifest refraction spherical equivalent; POD 2 m, Postoperative day 2 months; SE, spherical equivalent. *, *p* < 0.05 between groups. Target refractive values of Eyhance ICB00 group before and after surgery were −0.03 ± 0.16 and 0.15 ± 0.75, respectively. Target refractive values of ZCB00 group before and after surgery were −0.12 ± 0.37 and −0.38 ± 0.74, respectively

The target manifest refraction spherical equivalent (predicted power) before surgery was −0.03 ± 0.16 D for the Eyhance ICB00 group and −0.12 ± 0.37 D for the ZCB00 group. At two months postoperatively, manifest refraction spherical equivalent was 0.15 ± 0.75 D in the Eyhance ICB00 group and −0.38 ± 0.74 D in the ZCB00 group. In each group, there was no significant difference between the target predicted refractive power before surgery and the manifest refraction spherical equivalent at two months after surgery. However, there was a significant difference between the manifest refraction spherical equivalent at two months after surgery in the Eyhance ICB00 group and the ZCB00 group. The postoperative refraction value was more hyperopic in the Eyhance ICB00 group, and the ZCB00 group was more myopic than the preoperative refraction value (Fig. 1).

### Postoperative visual acuity and refraction

In the Eyhance ICB00 group, UDVA (logMAR) (at 5 m) was measured to be 0.50 ± 0.35 before surgery, 0.04 ± 0.04 at one week, 0.05 ± 0.07 at one month, and 0.05 ± 0.05 at two months after surgery. In the ZCB00 group, it was measured to be 0.36 ± 0.42 before surgery and 0.04 ± 0.08, 0.03 ± 0.06, and 0.02 ± 0.03 at one week, one month, and two months after surgery, respectively. In both groups, UDVA was significantly increased at one week, one month, and two months after surgery compared to that before surgery (all *p* < 0.05) (Table 2).

**Table 2:** Postoperative visual outcomes

Parameter	Eyhance® ICB00	Tecnis® ZCB00	*P* value
**Postoperative 1 week**			
UDVA (LogMAR)	0.04 ± 0.04	0.04 ± 0.08	0.122
UIVA (LogMAR)	0.09 ± 0.15	0.20 ± 0.14	0.013^*^
UNVA (LogMAR)	0.33 ± 0.20	0.44 ± 0.20	0.122
**Postoperative 1 month**			
UDVA (LogMAR)	0.05 ± 0.07	0.03 ± 0.06	0.088
UIVA (LogMAR)	0.10 ± 0.12	0.22 ± 0.12	0.018^*^
UNVA (LogMAR)	0.32 ± 0.14	0.43 ± 0.21	0.088
**Postoperative 2 months**			
UDVA (LogMAR)	0.05 ± 0.05	0.02 ± 0.03	0.008^*^
UIVA (LogMAR)	0.12 ± 0.14	0.22 ± 0.14	0.048^*^
UNVA (LogMAR)	0.29 ± 0.18	0.49 ± 0.22	0.008^*^
**Postoperative refraction**			
Automated refraction (AR)	−0.58 ± 0.15	−0.17 ± 0.12	
Subjective refraction (SR)	−0.11 ± 0.08	−0.14 ± 0.10	
AR within ± 0.5 D (%)	85.7	89.3	0.32
AR within ± 1.0 D (%)	89.2	94.6	0.48

Results are reported as mean ± SD

^*^, *p* < 0.05 between groups

In the case of UIVA (logMAR) (at 66 cm), in the Eyhance ICB00 group, it was measured to be 0.09 ± 0.15, 0.10 ± 0.12, and 0.12 ± 0.14 at one week, one month, and two months after surgery, respectively. In the ZCB00 group, it was measured to be 0.20 ± 0.14, 0.22 ± 0.12, and 0.22 ± 0.14 at one week, one month, and two months after surgery, respectively. In the case of UNVA (logMAR) (at 33 cm), it was measured to be 0.33 ± 0.20, 0.32 ± 0.14, and 0.29 ± 0.18 in the Eyhance ICB00 group and 0.44 ± 0.20, 0.43 ± 0.21, and 0.49 ± 0.22 in the ZCB00 group at one week, one month, and two months after surgery, respectively. Automated refractions of Eyhance ICB00 and ZCB00 groups were −0.58 ± 0.15 and −0.17 ± 0.12, respectively. And subjective refraction of Eyhance ICB00 and ZCB00 groups were −0.11 ± 0.08 and −0.14 ± 0.10, respectively. Percentage of the postoperative manifest refraction spherical equivalent within ± 0.5 D and ± 1.0 D was 85.7 and 89.2 (%) in the Eyhance ICB00 group and 89.3 and 96.4 in the ZCB00 group (Table 2).

**Fig. 2:**
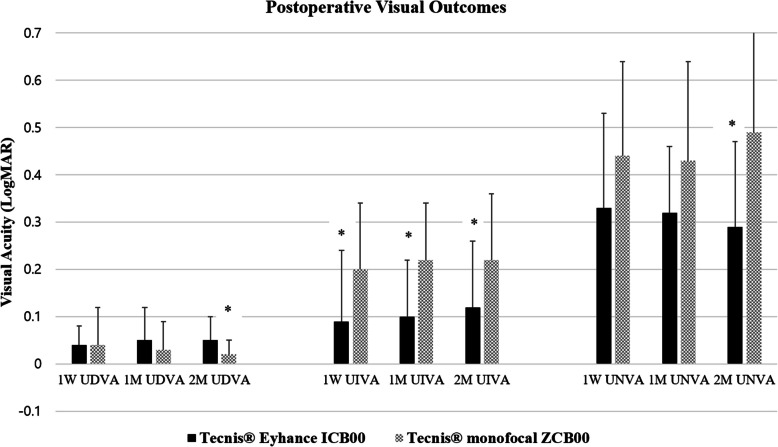
Postoperative visual outcomes. *, *p* < 0.05 between groups. One-week, One-month, and Two-month postoperative UDVA, UIVA, and UNVA in patients implanted with Tecnis® Eyhance ICB00 or Tecnis.® ZCB00

There was no significant difference in distance visual acuity at one week or one month postoperatively between the Eyhance ICB00 group and the ZCB00 group. However, at two months, UDVA in the ZCB00 group was significantly higher than that in the Eyhance ICB00 group (*p* < 0.05). At one week, one month, and two months after surgery, the UIVA of the Eyhance ICB00 group had significantly higher than that of the ZCB00 group (*p* < 0.05). There was no significant difference in near visual acuity between the two groups at one week or one month after surgery. However, at two months, the UNVA of the ICB00 group had significantly higher than that of the ZCB00 group (*p* < 0.05) (Table 2, Fig. 2).

### Defocus curve

**Fig. 3:**
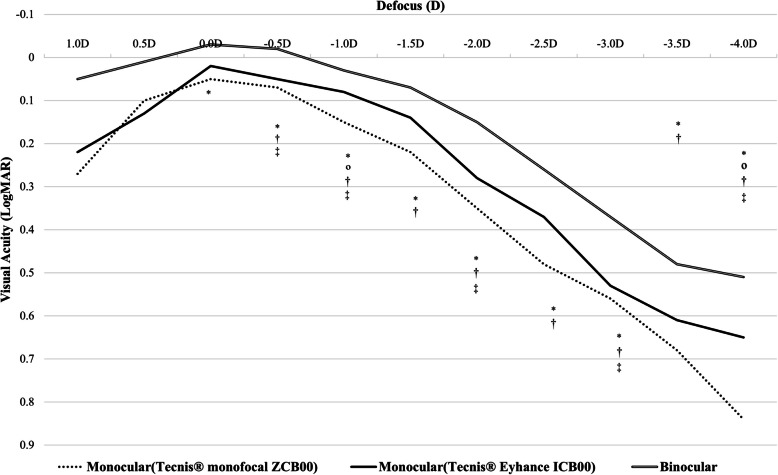
Mean binocular and monocular(Tecnis® Eyhance ICB00 or Tecnis® monofocal ZCB00) defocus curve. ^*^, *p* < 0.05 between three groups (binocular, monocular-Eyhance ICB00, ZCB00) by Kruskal Wallis test. º, *p* < 0.05 between two groups (Eyhance ICB00, ZCB00) by Mann–Whitney test. ^†^, *p* < 0.05 between two groups (binocular, ZCB00) by Mann–Whitney test. ^‡^, *p* < 0.05 between two groups (binocular, Eyhance ICB00) by Mann–Whitney test

Visual acuity was measured at a distance from + 1 D to −4 D, replacing a spherical lens in units of 0.5 D. A defocusing curve was obtained using the result. The depth of focus was 2.5 D in the ZCB00 group and 3 diopters in the Eyhance ICB00 group. The visual acuity of the Eyhance ICB00 group was better than that of the ZCB00 group in all ranges when a −0.5 diopter to −4 diopter spherical lens was added. However, the Eyhance ICB00 group had statistically significantly higher visual acuity than the ZCB00 group only when −1 D and −4 D spherical lenses were added (*p* < 0.05). In addition, when binocular visual acuity of a person with Eyhance ICB00 in one eye and ZCB00 in the other eye and monofocal visual acuity in the eye with Eyhance ICB00 or ZCB00 were compared in both the Eyhance ICB00 group and the ZCB00 group, binocular visual acuity was better than monocular visual acuity in all ranges. When binocular visual acuity and the monocular visual acuity of the Eyhance ICB00 group were compared, the binocular visual acuity significantly higher than that of the Eyhance ICB00 group when −0.5, −1, −2, −3 −4 D spherical lenses were added. When the binocular acuity and the monofocal visual acuity of the ZCB00 group were compared, the binocular visual acuity was statistically significantly better than that of the monofocal visual acuity when −0.5 to −4 D spherical lenses were added (Fig. 3).

Absolute and relative DOF of the Eyhance ICB00 group (1.15 ± 0.35, 1.47 ± 0.48 D) were significantly higher than those of the ZCB00 group (0.75 ± 0.26, 1.08 ± 0.38 D) (*p* < 0.05, respectively) (Table 3). There were no statistically significant differences in contrast sensitivity between the two IOLs for any contrast with or without glare (*p* > 0.05) (Table 4).

**Table 3:** Depth of focus values after surgery

**Parameter**	**Eyhance**®** ICB00**	**Tecnis® ZCB00**	***P* value**
Absolute DOF	1.15 ± 0.35	0.75 ± 0.26	0.01^*^
Realtive DOF	1.47 ± 0.48	1.08 ± 0.38	0.01^*^

Results are reported as mean ± SD

Absolute DOF (Depth of focus): vergences (in D) which provided VA values ≤ 0.1 logMAR

Relative DOF (depth of focus): vergences (in D) which provided a decay of 0.1 logMAR from to the best VA of each subject at zero vergence

Absolute and relative DOF of the Eyhance ICB00 group were significantly higher than those of the ZCB00 group (*p* < 0.05, respectively)

^*^, *p* < 0.05 between groups

**Table 4:** Contrast sensitivity after surgery

**Parameter**	**Eyhance**®** ICB00**	**Tecnis**®** ZCB00**	***P* value**
E100 (logMAR)	0.15 ± 0.10	0.13 ± 0.09	0.068
E25 (logMAR)	0.32 ± 0.18	0.31 ± 0.17	0.135
D100 (logMAR)	0.04 ± 0.04	0.03 ± 0.03	0.077
D25 (logMAR)	0.27 ± 0.16	0.25 ± 0.18	0.342
E100G (logMAR)	0.19 ± 0.12	0.17 ± 0.15	0.268
D100G (logMAR)	0.12 ± 0.08	0.11 ± 0.07	0.472

*E* Evening, *D* Day, *100* 100% contrast, *G* With glare

There were no significant differences of contrast visual acuity in both groups

^*^P < 0.05 compared to severe group

### Internal higher-order aberrations

Primary spherical aberration under 5 mm and 3 mm pupil size in the Eyhance ICB00 group (−0.21 ± 0.07, −0.04 ± 0.02 μm) were significantly greater negative than those in the ZCB00 group (−0.15 ± 0.04, −0.02 ± 0.01 μm) (*p* < 0.05, respectively). There were no significant differences of total higher-order, coma, and trefoil aberrations under 5 mm and 3 mm pupil size in both groups (*p* > 0.05) (Table 5).

**Table 5:** Internal higher-order aberrations after surgery

**Parameter**	**Eyhance**®** ICB00**	**Tecnis**®** ZCB00**	***P* value**
**5 mm pupil**			
Total HOAs	0.31 ± 0.12	0.30 ± 0.10	0.254
Coma	0.17 ± 0.05	0.16 ± 0.04	0.316
Primary SA	−0.21 ± 0.07	−0.15 ± 0.04	*0.02
Trefoil	0.18 ± 0.06	0.16 ± 0.05	0.429
**3 mm pupil**			
Total HOAs	0.10 ± 0.03	0.09 ± 0.03	0.435
Coma	0.05 ± 0.02	0.04 ± 0.02	0.278
Primary SA	−0.04 ± 0.02	−0.02 ± 0.01	*0.02
Trefoil	0.05 ± 0.02	0.05 ± 0.03	0.389

*HOA* Higher-order aberration, *SA* spherical aberration (μm)

Primary spherical aberration under 5 mm and 3 mm pupil size in the Eyhance ICB00 group were significantly greater negative than those in the ZCB00 group (*p* < 0.05, respectively)

^*^P < 0.05 compared to severe group

### Questionnaire

**Fig. 4:**
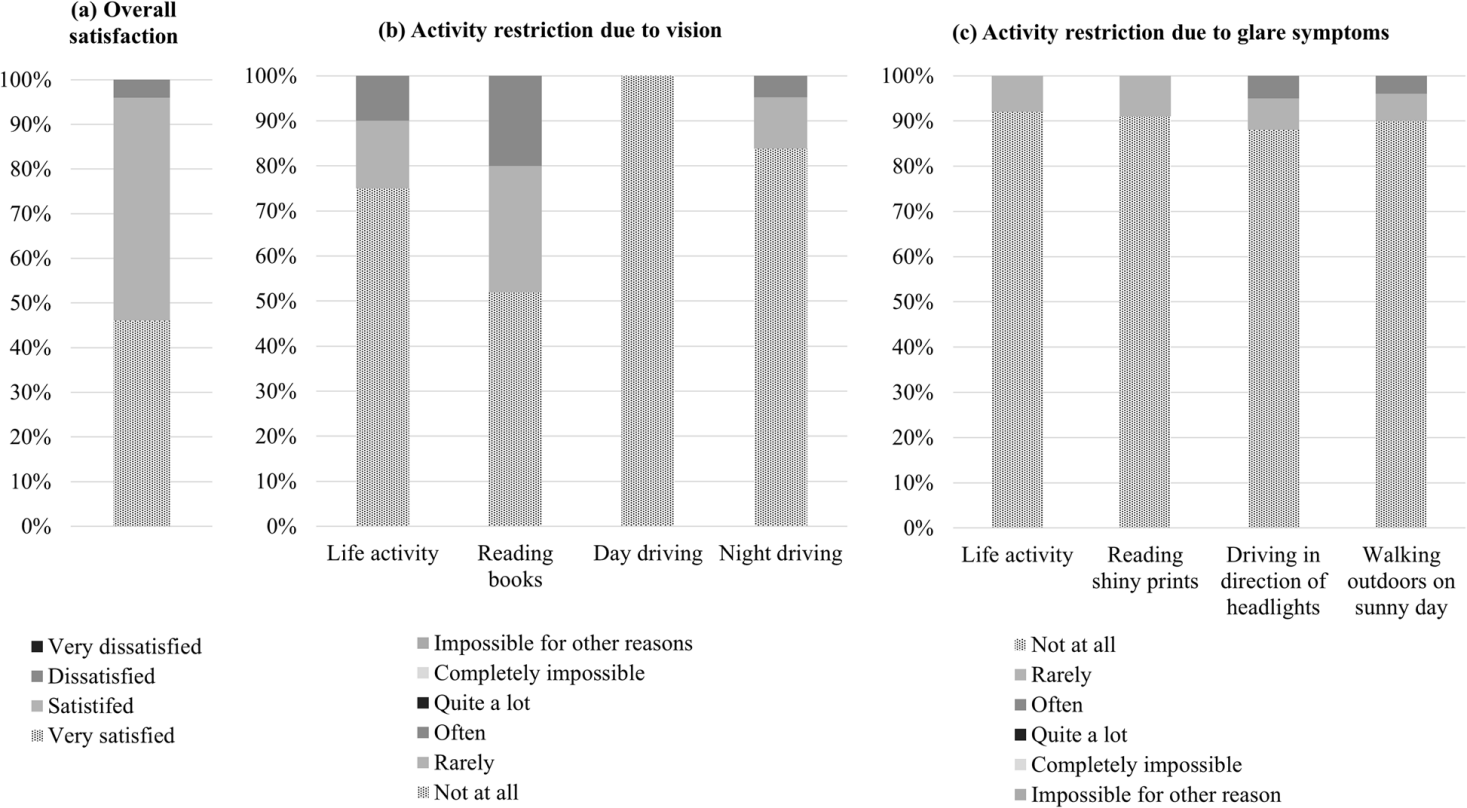
VRQOL-Cataract TyPE Spec questionnaire. Results of (**a**) overall satisfaction, **(b**) activity restriction due to vision, (**c**) activity restriction due to glare symptoms after implantation of Eyhance ICB00 and monofocal ZCB00 are shown

In order to find out the satisfaction and discomfort after surgery, a survey was conducted using the VRQOL-Cataract TyPE Spec questionnaire. The majority (96%) of respondents answered that they were satisfied with the overall satisfaction with the surgical result. When asked to what extent their eyesight limited or made it impossible to perform activities of daily living, reading, daytime driving, and night driving, 75%, 52%, 100%, and 88% of respondents answered that they were not restricted at all, and 15%, 28%, 0%, and 12% answered that they were a little limited, respectively. When asked how much glare restricted their activities of daily living, reading shiny print, driving towards headlights, and walking outdoors on a sunny day, 92%, 91%, 88%, and 90% answered that they were not at all restricted, respectively (Fig. 4).

## Discussion

When cataract surgery is decided, the patient is given a sufficient explanation about the type of intraocular lens. The patient then decides which intraocular lens to insert. Monovision has been tried to address the near vision difficulty after bilateral implantation of monofocal intraocular lenses [9–12]. Even monovision with a monofocal IOL in one eye and a multifocal IOL in the other eye has been reported [11, 13–15]. Therefore, we aimed to evaluate visual outcomes and patient satisfactions after implanting a monofocal IOL in one eye and a high aberration aspheric IOL in the contralateral eye in this study. Binocular visual acuity was confirmed through a defocus curve.

The Eyhance ICB00 group showed better results of UIVA, although the spherical equivalent was about 0.53D myopic change in the monofocal intraocular lens implanted eye compared to the high aberration aspheric intraocular lens implanted eye at two months postoperatively. This is thought to be because the Eyhance ICB00 is a high-aberration aspherical intraocular lens designed to have a continuous focus effect that can increase the depth of focus toward the center of the lens [16, 17]. There was no significant difference in near uncorrected visual acuity between the two groups at one week and one month after surgery. However, at two months after surgery, it was significantly higher in the Eyhance ICB00 group than in the ZCB00 group. Through this, it was found that the Eyhance ICB00 group had better near vision and intermediate distance vision than the ZCB00 group (Table 2).

There was difference between autorefraction and subjective refraction according to different types of IOLs. With monofocal IOLs, the autorefraction is in good consistency with the subjective with differences far below 0.5 diopter (D) for the spherical equivalent. But the difference between autorefraction and subjective refraction was larger than 1.0 D with multifocal IOLs and −0.85 D with EDoF IOLs [18]. In our study, there was little difference between autorefraction and subjective refraction with monofocal IOLs, but the difference between autorefraction and subjective refraction was −0.47 D with enhanced monofocal IOLs (Table 2).

Our study is in alignment with previous reports that the Eyhance ICB00 lens demonstrates greater depth of focus than its monofocal counterpart. There was no statistically significant visual acuity difference in the defocus curve except when −1 D and −4 D spherical lenses were added. However, when spherical lenses between 0 and −4 D were added, the visual acuity of the Eyhance ICB00 group was better than that of the ZCB00 group in all ranges. In other words, Eyhance ICB00 group have good intermediate VA, but it also have excellent far to intermediate VA (−1 D: 1 m) and very near VA (−4 D: 25 cm) compared to ZCB00 group in this study. In addition, it was confirmed that binocular vision was better than that of monocular acuity. In particular, when comparing the binocular visual acuity of a person with Eyhance ICB00 in one eye and monofocal ZCB00 in the other eye and monocular visual acuity in the eye with Eyhance ZCB00, when a −0.5 D to −4 D spherical lens was added, the binocular visual acuity was significantly better than the monocular visual acuity. For binocular vision, even one eye would have improved intermediate distance vision with an Eyhance ICB00 intraocular lens. In addition to the effect of Eyhance ICB00, it was thought that the visual acuity was 10–20% better than that of monocular due to binocular summation in general (Fig. 3).

In the questionnaire conducted after surgery, most patients answered that their daily activities were not limited by their vision or glare. Monofocal IOLs and high-aberration aspherical IOLs have fewer symptoms such as glare and halo that can occur with multifocal IOLs. In particular, in the case of high-order aspheric IOLs, due to the design without the diffraction ring or refractive zone used in the multifocal IOL, it is thought that the intermediated visual acuity can be improved with less halo and glare compared to the multifocal IOL [19].

Intraindividual comparison of an enhanced monofocal and an aspheric monofocal IOL of the same platform was also reported [19]. Significantly increased monocular DCIVA at 80 cm and 66 cm and DCNVA at 40 cm were observed with the enhanced ICB00 IOL, and the ZCB00 IOL demonstrated better BCDVA but uncorrected VA measurements were not performed in this study [19]. In our study, we included uncorrected VA measurements.

The ZCB00 group had higher postoperative UDVA than in the Eyhance ICB00 group, but the Eyhance ICB00 group had higher postoperative UIVA than in the Eyhance ICB00 group in this study.

Many people are interested in the quality of vision and visual acuity after cataract surgery. IOLs can be used semi-permanently unless there are special complications. It is important to select the IOL well in the first place because replacing an IOL that has already been inserted has a high risk. Therefore, ophthalmologists must understand and utilize the rapidly changing and developing characteristics of the intraocular lens to maintain the highest possible quality of life after cataract surgery.

The ICB00 group has significantly greater negative primary internal spherical HOAs(SAZ(4,0)) compared to the ICB00 group [20]. The internal and ocular SAZ(4,0) were decreasing from larger to smaller pupil sizes [20]. In our result, primary spherical aberration under 5 mm and 3 mm pupil size in the Eyhance ICB00 group (−0.21 ± 0.07, −0.04 ± 0.02 μm) were significantly greater negative than those in the ZCB00 group (−0.15 ± 0.04, −0.02 ± 0.01 μm) (*p* < 0.05, respectively) (Table 5).

The first limitation of this study was that the number of subjects was small, 56 in each group. Thus, a follow-up study with more patients is needed in the future. Second, the follow-up period after surgery was as short as two months. In the case of general cataract surgery, it is rare that long-term follow-up is required, as most patients reach a clinically stable state during the two-month follow-up period. Considering the situation that requires change and adaptation after inserting different intraocular lenses in the same patient, a long-term follow-up will be necessary. The third limitation of this study was study design. Using dominant eyes implanted with ZCB00 IOL to form a group and the fellow eyes implanted with ICB00 to form the second group limits the advantages of having implanted the same patient with 2 different IOLs. Monofocal IOLs usually have excellent visual performance aimed for distant vision, high contrast sensitivity, and low rates of photic adverse effects [21]. Therefore, the ZCB00 IOL was implanted in the dominant eye and the ICB00 IOL in the nondominant eye. The forth limitation was that there was no comparison with a control group of patients who underwent bilateral implantation of a monofocal IOL like earlier report [21]. We think this paper can be a good reference for uncorrected visual quality after implantation of monofocal IOL on one eye and higher-order aspheric IOL on the other eye because we analyzed uncorrected near, intermediate, and distant VA unlike earlier report [21] only including corrected vision.

## Conclusion

The intermediate distance visual acuity of the Eyhance ICB00 group was significantly better than that of the ZCB00 group. The binocular visual acuity of a person with Eyhance ICB00 in one eye and ZCB00 in the other eye was also superior to the ZCB00 monocular acuity. Through this, it is considered that inserting a high-order aspherical IOL at one eye is better for improving both distance and intermediate distance vision than inserting monofocal IOLs in both eyes. In addition, there is no discomfort in daily life in patients with Eyhance ICB00 inserted into a single eye.

## Data Availability

The datasets used and/or analyzed during the current study available from the corresponding author on reasonable request.
